# Knowledge and Level of Awareness Regarding Breast Cancer and Practices of Breast Screening Methods Among Female Riyadh Citizens

**DOI:** 10.7759/cureus.59996

**Published:** 2024-05-09

**Authors:** Areej A Alhumaid, Waad Alshahrani, Shuruq M Al Qahtani, Hawazin Alotaibi, Ruba A Almubarriz

**Affiliations:** 1 General Surgery, Breast Surgical Oncology, King Abdullah Bin Abdulaziz University Hospital, Riyadh, SAU; 2 Medicine, Princess Nourah Bint Abdul Rahman University, Riyadh, SAU; 3 General Practice, Ministry of Health Holdings, Riyadh, SAU

**Keywords:** breast self-examination, mammogram screening, awareness, knowledge, breast cancer

## Abstract

Background

Breast cancer is the most common cancer in women. Some of the risk factors for breast cancer include family history, personal history, and hormonal replacement therapy. There are different methods of screening breast cancer, including clinical examination, breast self-examination (BSE), and mammograms. Despite the various modalities of screening, the screening was low, and the level of awareness was variable.

Objective

The objective of this research is to determine the knowledge and level of awareness regarding breast cancer and BSE among female Riyadh citizens.

Methods

A cross-sectional study was conducted on 408 participants, using a convenient sample technique. The inclusion criteria were female, Riyadh residents, aged between 18 and 70 years old. An online survey was distributed among female Riyadh residents, and it included demographic data, knowledge regarding breast cancer, its risk factors, BSE, and mammograms. The questionnaire was constructed for this study, and validity and reliability were tested. Statistical Product and Service Solutions (SPSS, version 22; IBM SPSS Statistics for Windows, Armonk, NY) software was used for statistics. Analysis of quantitative data by a t-test and association of qualitative variables by a chi-square test was conducted. A P-value less than 0.05 was considered statistically significant.

Results

A total of 408 participated in our sturdy, with the majority aged 50 years and above. All of those aged between 18 and 30, in addition to the majority of those aged 50 and above, showed poor knowledge. Gathering information through campaigns and TV/radio was associated with better knowledge compared to other sources. Knowledge regarding breast cancer risk factors was better among younger participants compared to older ones. Regarding BSE, all participants, regardless of age, showed poor knowledge. However, older participants aged 50 and above showed good knowledge in regard mammograms.

Conclusion

Most of the participants showed poor knowledge in regards to breast cancer symptoms and signs, risk factors, and BSE. However, knowledge regarding mammograms was mostly good among the participants. The variations in knowledge were influenced by several factors, including age, educational level, number of kids, information sources, and prior history of history of breast cancer or of a relative.

## Introduction

According to the World Health Organization (WHO), the most common cancer worldwide as of the end of 2020 was breast cancer, which had been diagnosed in 7.8 million women in the previous five years [1]. The global estimate suggests nearly 10 million deaths of cancer worldwide in 2020, making it the leading cause of death, with 685,000 deaths worldwide in 2020 due to breast cancer [1]. According to a Saudi Cancer Registry data on incidence from 2017, breast cancer was the most common cancer among women and accounted for 18.1% of all cancers. Among women of all ages, breast cancer accounts for 30.9% of all cases of cancer reported, with a median age of 51 years at the time of diagnosis [2].

Family history and personal history of breast cancer, chest radiation, smoking, alcohol, hormonal replacement therapy, aging, late menopause, high-fat diet, lack of physical activity, never breastfed, contraceptives, late age at full-term pregnancy, and obesity are risk factors of breast cancer [1-3]. The warning signs and symptoms of breast cancer are breast lump, changes in breast size and shape, pain in the breast or armpit, bloody or fluid nipple discharge, redness of breast skin, changes in nipples, lump or fullness in the armpit, dimpling of the breast skin, rash or ulcer on the nipple, and arm swelling [1-3]. A study conducted in the United Arab Emirates at the University of Sharjah, published in 2019, on 241 female students shows that 49.8% of the participants had some understanding of the risk factors for breast cancer. The most commonly identified risk factors were family history of breast cancer and personal history of breast cancer with 84.2% and 82.2%, respectively. Followed by smoking at 63.1% and chest radiation at 59.3%. On the other hand, the least identified risk factors were obesity and advanced maternal age at full-term pregnancy with 25.7% for both. The majority of the participants (61.8%) lacked awareness of breast cancer warning signs and symptoms, whereas only 38.2% did so. The most identified warning signs and symptoms of breast cancer were breast lump at 80.1%, changes in breast size and shape at 74.7%, and pain in the breast or armpit at 73.3%. The least common breast cancer warning signs and symptoms were arm swelling and a rash or ulcer on the nipple 37.8% and 26.1%, respectively [3].

The likelihood of successfully treating cancer is considerably increased by early identification. The two elements of early cancer detection are screening and early diagnosis. While screening consists of evaluating healthy individuals to find those who have cancer before any symptoms occur, early diagnosis focuses on identifying symptomatic patients as early as feasible [4]. BSE is one of the breast cancer screening methods that can be performed at home, as it is an easy, simple, and effective procedure that women may perform without the help of a specialist. It does not require specific equipment or a hospital visit. Although the American Cancer Society does not recommend regular BSE, whether performed by women themselves or by medical professionals (clinical breast exams) has not been proven to have any significant benefits. There is very little proof that, when women additionally have screening mammography, these tests aid in the early detection of breast cancer. Most of the time, when breast cancer is discovered due to symptoms such as a lump in the breast, a woman finds the symptom while doing routine tasks such as taking a shower or getting dressed. Although the American Cancer Society still encourages women to be aware of how their breasts typically feel and seem and they should notify a healthcare professional of any changes as soon as possible [5]. However, research conducted in primary healthcare in Najran showed that approximately half of the participants demonstrated a low level of BSE (56.8%) [6]. Another study at King Saud University demonstrates that the majority of students in the third level (67.2%) had a higher knowledge level, compared to more than half of the participants in the second level (61.3%) who had a sufficient understanding of BSE and breast cancer. Only 18% of students have BSE, while 81% of participants do not [7].

Mammograms have been the cornerstone in the detection of breast cancer in its early stages and in reducing mortality [8]. As for the higher incidence of breast cancer in Saudi Arabia, the Ministry of Health recommended to start screening using mammograms at the age of 40 and to be repeated every two years until the age of 50 and then annually [9]. However, a study that was conducted in Madinah City showed poor utilization of mammograms and other modalities of screening [8]. A study that was conducted in 20 primary care centers and four private hospitals in Riyadh reported that only 10% of the participants aged 40 and above had done mammograms [10]. In a study that was conducted in Madinah, the barriers to taking mammograms were that they believed it was a painful procedure or it would expose them to more radiation. These were found to reduce the use of mammograms by 56% and 48%, respectively [8]. In recent studies, it was linked that low socioeconomic status, low education level, and lack of knowledge regarding available methods of screening would be associated with low intake of mammograms [9].

## Materials and methods

This is a cross-sectional-based study using a non-probability convenience sample. An ethical approval letter has been acquired from the Research Ethics Committee, Princess Noura Bint Abdulrahman University, Kingdom of Saudi Arabia, prior to this study. The anonymity of all the participants in this study was ensured. An electronic survey was conducted among Riyadh residents of Saudi Arabia of those aged 18-70 years old females who are willing to participate in the study. We aimed to investigate their knowledge and level of awareness regarding breast cancer and practices of breast cancer screening methods.

The questionnaire was designed using Research Electronic Data Capture (REDCap). This questionnaire was taken from the breast cancer awareness measure developed by Cancer Research UK, King’s College London, and University College London in 2009 [11]. Then, it was translated into Arabic by one researcher who did not read or see the English version, and we checked the Arabic version with an independent translator who is an expert in the Arabic language. Next, it was re-translated into English and was compared with the original questionnaire. It also gets reviewed by an expert breast surgery consultant. After that, both the reliability and the validity of the Arabic version were assessed in 10 people in Riyadh, Saudi Arabia. After that, both the reliability and the validity of the Arabic version were assessed in 10 people from the general population in Riyadh, Saudi Arabia. Then, we got their feedback regarding the questionnaire, and they did not have any questions or comments about the questionnaire.

The link was shared on social media, such as WhatsApp, Instagram, and Twitter from December 2022 to January 2023.

Our minimal sample size was 384 calculated using OpenEpi (version 3; Centers for Disease Control and Prevention, Atlanta, GA) with a 95% confidence interval (alpha=0.05) and with a power study of 95% (beta=0.05).

The questionnaire consisted of five sections. The first demographic data included age, nationality, marital status, number of children, level of education, and job status. The second section is about knowledge regarding breast cancer in general. In the last three sections, we measure the knowledge regarding breast cancer risk factors, knowledge, and practice of BSE and lastly knowledge regarding mammograms. We analyzed the results using a scoring system, and the data were divided into two groups: good knowledge and poor knowledge. Those who got 60% total or above of right answers in each section were considered to have good knowledge, while those who had less were considered to have poor knowledge.

Statistical Product and Service Solutions (SPSS, version 26; IBM SPSS Statistics for Windows, Armonk, NY) software was used for data analysis: we described the variable in mean ± SD or percentage as appropriate. The chi-square test was used to determine the statistical significance between the two variables. A P-value less than 0.05 was considered statistically significant.

## Results

Table 1 shows the demographic data of the participants. Out of 408, 47 were aged between 18 and 30, 69 were between 31 and 40, 114 aged between 41 and 50, and 174 aged above 50 years. Regarding marital status, the majority of the participants (77%) were married, and the minority (2.7%) were widows. Moreover, 58.1% had a bachelor's degree, and 27% had completed either middle or high school. The majority were unemployed with 36.8%, 32.8% were employed, and 2.7% had private business.

**Table 1:** Characteristics of the study sample (n=408)

Items	Statistics
Age (years)	
18-30	47 (11.5%)
31-40	69 (16.9%)
41-50	114 (27.9%)
>50	175 (42.9%)
Nationality	
Saudi	401 (98.3%)
Non-Saudi	7 (1.7%)
Current Residency	
Riyadh city	364 (89.2%)
Outside Riyadh city	43 (99.8%)
Marital status	
Single	49 (12%)
Married	314 (77%)
Divorced	34 (8.3%)
Widow	11 (2.7%)
Kids	
No kids	68 (16.7%)
One or two kids	78 (19.1%)
Three or more kids	262 (64.2%)
Educational level	
Middle school	37 (9.1%)
High school	73 (17.9%)
Diploma	38 (9.3%)
Bachelors	237 (58.1%)
Masters or Ph.D	23 (5.6%)
Occupation	
Student	20 (4.9%)
Unemployed	150 (36.8%)
Retired	93 (22.8%)
Private business	11 (2.7%)
Employed	134 (32.8%)
Heard about breast cancer	
Yes	11 (2.7%)
No	396 (97.3%)
Knowledge source	
Health care workers	85 (20.8%)
Campaigns	249 (61%)
Books, magazines or news	131 (32.1%)
TV/Radio	132 (32.4%)
Social media	213 (52.2%)
I don’t have information regarding breast cancer	24 (5.9%)
Previously diagnosed with breast cancer	
Yes	47 (11.6%)
No	357 (88.4%)
Relative diagnosed with breast cancer	
Yes	152 (37.4%)
No	254 (62.6%)

Additionally, 97.3% of the participants have not heard about breast cancer, while 2.7% have heard about it.

The majority of the participants received information in regard to breast cancer from campaigns, followed by social media, while receiving information from healthcare workers was the least source.

Knowledge of breast cancer symptoms and signs

Most of the participants had poor knowledge regarding breast cancer signs and symptoms. Moreover, 100% of the age group 18-30 scored less than 60% and thus were considered to be poor knowledge. However, of those who are 50 years and older, 12% of them had good knowledge as they scored 60% and above with a p-value of 0.023. Our study showed that women with no kids had 8.8% good knowledge; in comparison with women who had one or two kids, 20% had good knowledge. Regarding the source of information, campaigns and TV/radio were the highest among the other sources regarding good knowledge of the signs, and symptoms nearly 15% of them were knowledgeable.

Knowledge of breast cancer risk factors

**Figure 1:**
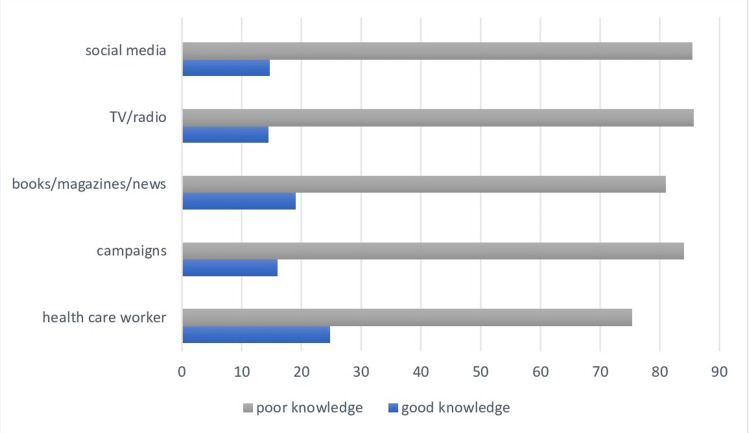
Source of information regarding breast cancer risk factors

Our study showed that one-third of the participants in the age group 18-30 had good knowledge of breast cancer risk factors. This percentage decreased in the older age group, as shown in Table 2, and more than 85% of women who are 50 years and older have poor knowledge of breast cancer risk factors. The majority (90%) who chose healthcare workers as a source of information had poor knowledge. Additionally, 88% who chose health campaigns had poor knowledge regarding risk factors, as shown in Figure 1. 

**Table 2:** Level of knowledge regarding breast cancer risk factors in different age groups

Items	Good knowledge	Poor knowledge	P-value
Age (years)			
18-30	15 (31.9%)	32 (68.1%)	0.014
31-40	12 (17.3%)	57 (82.7%)	
41-50	15 (13.1%)	99 (86.9%)	
>50	23 (13.1%)	152 (86.9%)	

BSE

Regarding the knowledge of BSE, all the participants with different age groups showed poor knowledge about BSE. Additionally, 99.1% of the age group 41-50 had poor knowledge, which is the highest compared to other groups. Regarding marital status, 100% of studied women who were divorced, or widowed had poor knowledge. On the other hand, single women present the lowest percentage of poor knowledge (95.9%). Most of the women with two or fewer kids showed poor knowledge reaching 98.7%.

Referring to education level, 100% of high school women had poor knowledge, 94.4% of middle school women showed poor knowledge, and bachelor's degree women (97.8%) showed poor knowledge. However, 100% of participants were employed, and they had poor knowledge regarding BSE.

**Figure 2:**
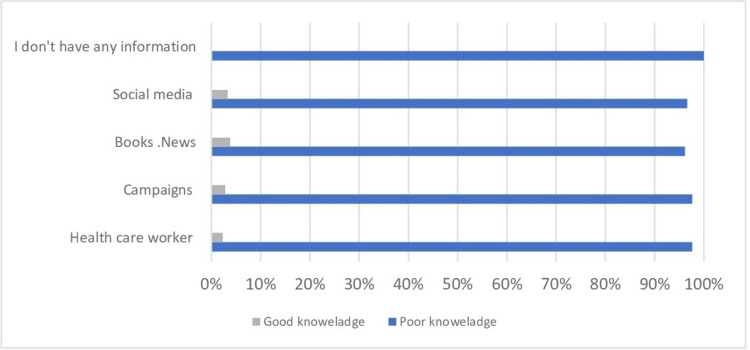
Source of information regarding breast self-examination

Figure 2 shows the source of information of the participants. Specifically, 97% of participants who chose campaigns and healthcare workers as a source of their information showed poor knowledge.

More than half of the participants performed BSE though 96.8% had poor knowledge.

Mammogram screening

Table 3 presents the knowledge regarding mammogram screening. Most of the participants showed good knowledge, with 80.5% of participants aged above 50, 76% between 31 and 50 years, and 63% between 18 and 30 years.

**Table 3:** Level of knowledge regarding mammogram screening

Items	Good knowledge	Poor knowledge	P-value
Age (years)			
18-30	29 (63.04%)	17 (36.95%)	0.095
31-40	51 (75%%)	17 (25%)	
41-50	87 (76.99%)	26 (23.0%)	
>50	141 (80.57%)	34 (19.42%)	
Marital status			
Single	32 (65.3%)	17 (34.69%)	0.197
Married	245 (78.77%)	66 (21.22%)	
Divorced	25 (73.52%)	9 (26.47%)	
Widow	9 (81.81%)	2 (18.18%)	
Kids			
No kids	44 (64.70%)	24 (35.29%)	0.028
One or two kids	58 (76.31%)	18 (23.68%)	
Three or more kids	209 (80.07%)	52 (19.92%)	
Educational level			
Middle school	30 (81.08%)	7 (18.91%)	0.890
High school	55 (76.38%)	17 (23.61%)	
Diploma	27 (71.05%)	11 (28.94%)	
Bachelors	181 (77.02%)	54 (22.97%)	
Masters or ph.D	18 (78.26%)	5 (21.73%)	
Occupation			
Student	11 (55%)	9 (45%)	0.090
Unemployed	116 (78.91%)	31 (21.08%)	
Retired	68 (73.11%)	25 (26.88%)	
Private business	10 (90.90%)	1 (9.09%)	
Employed	106 (79.10%)	28 (20.89%)	
Previously diagnosed with breast cancer			
Yes	39 (84.78%)	7 (15.21%)	0.198
No	271 (76.33%)	84 (23.66%)	
Relative diagnosed with breast cancer			
Yes	125 (82.78%)	26 (17.21%)	0.025
No	184 (73.01%)	68 (26.98%)	
People who done a mammogram before	119 (92.96%)	9 (7.03%)	<0.001

Regarding marital status, the majority of studied women were knowledgeable, which is the highest amongst widow women with a percentage of 81.18%, followed by married women with 78.7%. This study also showed that women who had three or more kids had the highest percentage of good knowledge, reaching 80%, in comparison to women who had no kids, with a percentage of 64.7% (P=0.028).

Referring to education level, surprisingly, the most knowledgeable group were those with mid-school education, with 81% of them scoring as good knowledge. 

Most of the participants had good knowledge regarding mammogram screening.

**Figure 3:**
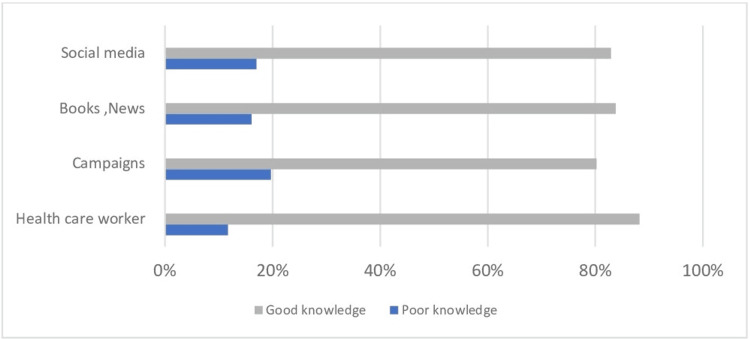
Source of information regarding mammogram screening

Figure 3 presents the source of knowledge regarding mammogram screening. The most common source of their knowledge was obtained from their healthcare worker (88.2%), whereas 83.2% from books/news, a total of 82.9% from social media, and 80.2% from campaigns, which is statistically significant (P<0.05).

A total of 84.7% who were previously diagnosed with breast cancer showed good knowledge of mammogram screening. Additionally, women who had relatives diagnosed with breast cancer showed a high percentage of good knowledge (82.7%), which is statistically significant (P=0.025).

Additionally, 92.96% of women who had mammograms had good knowledge regarding mammogram screening, which is statistically significant (P<0.001; Table 3).

## Discussion

Knowledge of breast cancer symptoms and signs

Most of the participants in our study had poor knowledge regarding breast cancer signs and symptoms. This was similar to the study done at ALSARJAH University; more than two-thirds of participants were non-knowledgeable about the warning signs and symptoms of breast cancer [3]. In addition to the study done in Madinah, 35% had poor knowledge, and 30% had fair knowledge regarding breast cancer clinical pictures [8]. 

Knowledge of breast cancer risk factors 

Our finding can be compared to a study done in Madinah, and more than half of the women had poor knowledge regarding breast cancer risk factors [8]. In contrast to a study done in Abha, more than half of the women identified breast cancer risk factors [12]. The majority of participants who chose healthcare workers and health campaigns as a source of information had poor knowledge. In contrast to the AL Sharjah study, participants attending the medical campus were knowledgeable about breast cancer risk factors 55.0% [3].

BSE

Our study showed a low level of knowledge regarding BSE. This is similar to a previous study in Najran showing that more than half of the participants demonstrated a low level of knowledge regarding BSEs [6]. In addition, a study done in Abha showed that less than half (41.5%) of participants have heard about BSE [12]. Meanwhile, another study done in Sharjah showed that more than half (68.9%) had heard about BSE [3]. In the present study, the main source of information regarding BSE was from healthcare workers and campaigns in contrast to the previous studies [3-6] showing that the main source was social media. Similar to a study that targeted students at King Saud University, the most common source of information was the mass media - TV [7].

Mammogram screening 

Our study showed good knowledge regarding mammogram screening, similar to the study done in North Saudi Arabia with 66.2% aware of the MOH Saudi Arabia’s recommendation for mammogram screening for breast cancer [9]. In contrast to the study done in Najran, which showed that more than 80% of women displayed a low level of mammogram knowledge [6], another study done in Abha showed that only 22% of women knew about mammograms [12]. In the present study, the main source of knowledge regarding mammograms was healthcare workers, in contrast to the study done in Najran, which showed that social media was the main source [6]. In our study, the majority of studied women who underwent mammograms had good knowledge, in contrast to the study done in Al Madinah, which showed that the studied women who underwent mammograms had poor and fair knowledge [8]. The limitation of this study is that the data were collected through an online REDCap form, as it is distributed through a particular group of people, rather than all types of people, such as the elderly.

## Conclusions

Breast cancer is the most common cancer among women. The awareness and knowledge among Riyadh female residents differed by different factors. These factors are age, marital status, educational level, occupation, prior personal diagnosis with breast cancer, and a relative diagnosed with breast cancer. The majority of the participants showed poor knowledge regarding breast cancer signs, symptoms, risk factors, and BSE. However, good knowledge of mammograms was noticed. The majority of those participants obtained their knowledge through healthcare workers. Thus, emphasis on utilizing breast awareness day campaigns, counseling at the clinics, and health education through social media platforms should encouraged. Additionally, additional studies are needed in the area of BSE to study the reasons for such low levels of knowledge about breast cancer with the aim of increasing breast awareness.
